# Reverse “cheese wire” fenestration for abdominal aortic dissection repair: a case report and literature review

**DOI:** 10.1186/s12893-022-01581-4

**Published:** 2022-04-21

**Authors:** Tonglei Han, Yani Wu, Chong Jin, Xiaolong Wei, Zhiqing Zhao

**Affiliations:** 1 Department of Vascular Surgery, Changhai Hospital, Second Military Medical University, 168 Changhai Road, 200433 Shanghai, China; 2 Department of Breast and Thyroid Surgery, Changhai Hospital, Second Military Medical University, 168 Changhai Road, Shanghai, 200433 China; 3 Department of General Surgery, Taizhou Central Hospital, Taizhou University Hospital, No. 999 Donghai Avenue, Taizhou, 318000 Zhejiang China

**Keywords:** Cheese wire, Fenestration, Endovascular repair, Aortic dissection

## Abstract

**Background:**

Aortic dissection is one of the most common emergency condition leading to internal organs or lower limb ischemia and aortic rupture. Herein, we described a reverse “cheese wire” endovascular fenestration repair (CWFER) in a patient with complicated abdominal aortic dissection which had never been reported.

**Case presentation:**

A 62-year-old male presented abdominal tear-like pain and acute ischemia of the right lower extremity during the endovascular treatment of celiac trunk aneurysms. Computed tomography angiography (CTA) and digital subtraction angiography (DSA) showed abdominal aortic type B dissection with acute ischemia of the right lower extremity preoperatively. After a detailed preoperative examination, the patient then was performed a reverse CWFER. So far, the patient has been followed-up for 6 months, postoperative CTA demonstrated good stent-graft expansion and perfusion of bilateral common iliac arteries; also, no endoleak was detected.

**Conclusions:**

The right iliac artery in this patient supplied by false lumen, which lead to acute ischemia of the right lower extremity, needed to be treated as an emergency and dealt with promptly. CWFER is a very high-risk treatment that requires the rich experience of vascular surgeon and accurate assessment of aortic dissection. After interventional treatment, the patient recovered uneventfully after 6 months’ follow-up.

**Supplementary information:**

The online version contains supplementary material available at 10.1186/s12893-022-01581-4.

## Background

B-type aortic dissection is one of the most common emergent conditions affecting the aorta [1–4]. Clinically, open surgery or percutaneous aortic fenestration is used for the correction of poor perfusion [5]. Although endovascular repair is considered as an optional method, the delivery and deployment of endograft into the constrained true lumen remain challenging. Herein, we described a reverse “cheese wire” endovascular fenestration repair (CWFER) in a patient with complicated abdominal aortic dissection.

## Case presentation

**Fig. 1:**
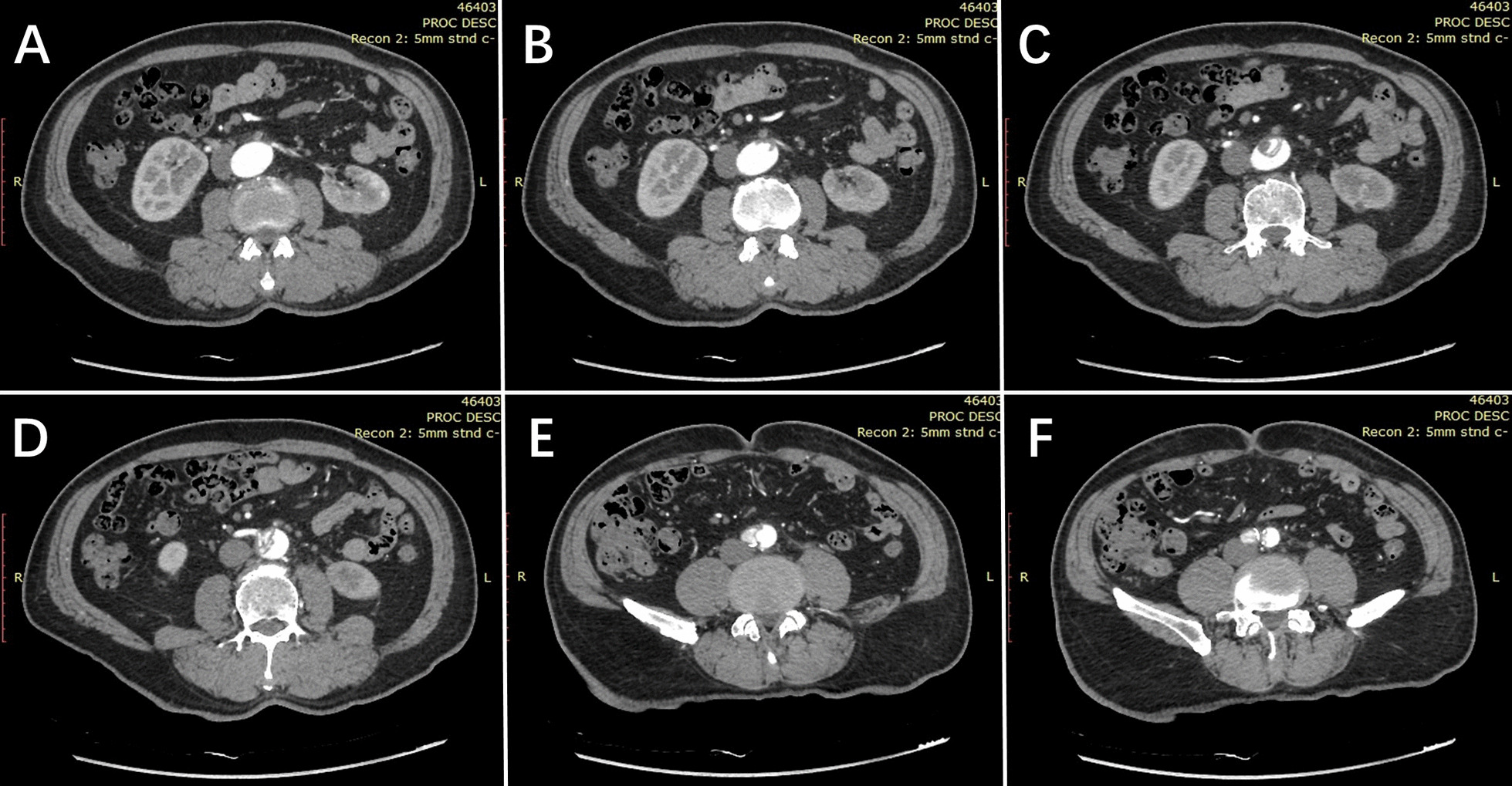
Preoperative axial-view surveillance computed tomography scans. **A** Normal aorta above the left renal ostia. **B** The proximal end of abdominal aortic dissection beneath the left renal ostia. **C** The middle part of the abdominal aortic dissection. **D** Inferior mesenteric artery supplied by the false aortic lumen. **E** The aorta above the bifurcation. **F** Right iliac artery supplied by false aortic lumen

**Fig. 2:**
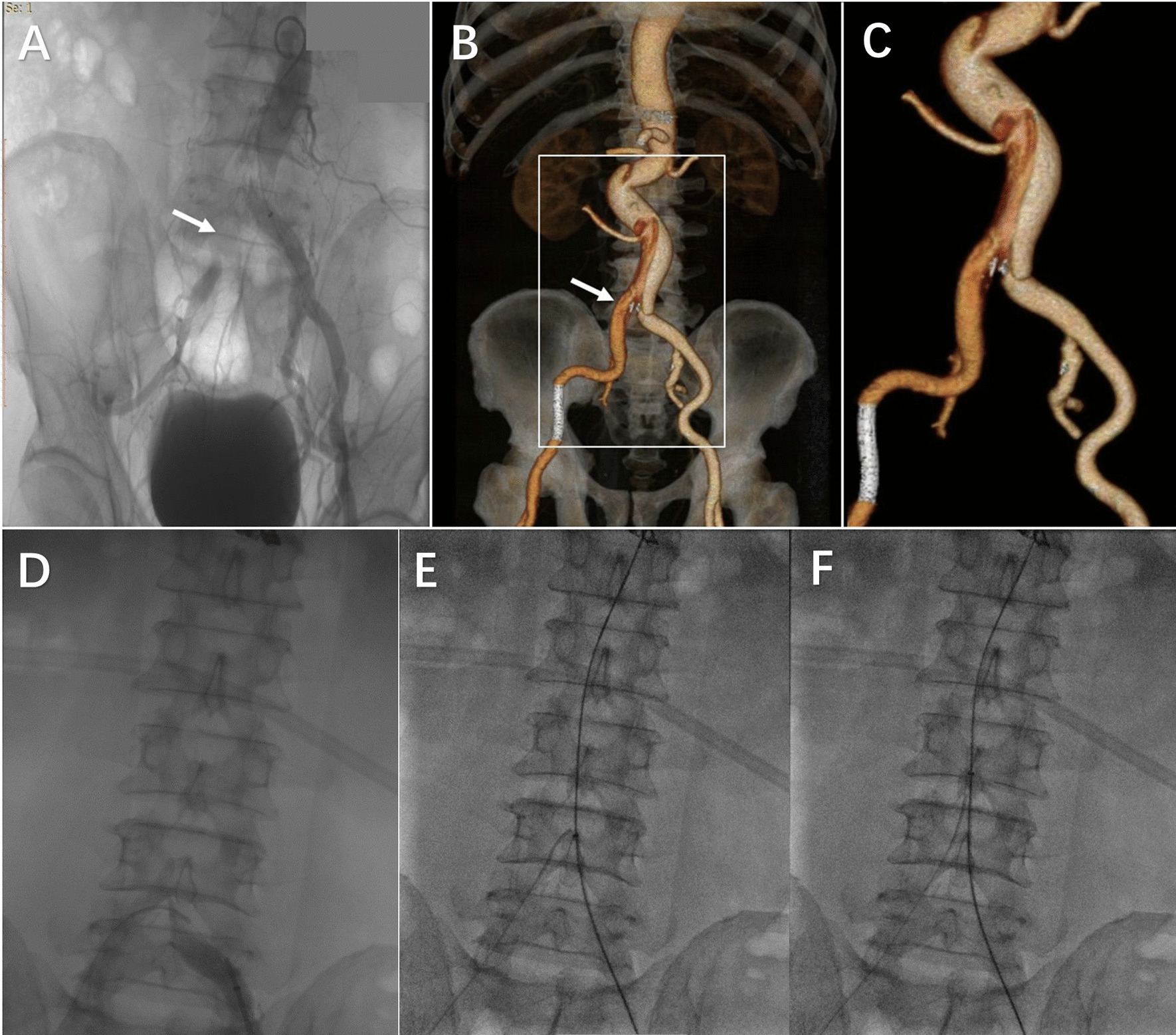
Preoperative digital subtraction angiography, three-dimensional reconstruction image, and instructions for “cheese-wire” technology. **A** Abdominal aorta angiography shows true lumen occlusion (white arrow) of the right common iliac artery. **B** The right common iliac artery (white arrow) is supplied by the false lumen. **C** Preoperative three-dimensional reconstruction of the abdominal aortic dissection. **D** The 0.035-inch guidewire (Terumo) was captured by the gooseneck snare, and the through-and-through guidewire control was obtained. **E** A 0.035-inch Lunderquist guidewire was advanced to the thoracic aorta from the long sheath in the left femoral artery. **F** Under V-18 guidewire tension, the guidewire was pulled upwards to shear the flap

A 62-year-old male presented abdominal tear-like pain and acute ischemia of the right lower extremity during the endovascular treatment of celiac trunk aneurysms. Computed tomography angiography (CTA) and digital subtraction angiography (DSA) showed abdominal aortic type B dissection with acute ischemia of the right lower extremity preoperatively (Figs. 1 and 2), and the primary entry tear was localized below the renal artery. The left iliac artery was originated from the true lumen, while the false lumen supplied the right iliac artery but could not be observed on imaging due to significant compression of the false lumen.

This patient provided prior informed consent to this intervention, as well as the publication of the relevant data. Anticoagulation therapy [low molecular weight heparin (LMWH), enoxaparin, 40 mg, SI, bid] was prescribed before the patient underwent the procedure. The endovascular repair of abdominal aortic dissection was performed under general anesthesia. The orientation of the puncture was guided by fluoroscopy. The percutaneous access of both femoral arteries was created by placing a 6 F short sheath (Terumo). The gooseneck snare was introduced into the false lumen of the lower abdominal aorta from the left femoral artery under the guidance of the path map. A 0.035-inch guidewire (Terumo) was introduced into the false lumen from the right femoral artery and captured by the gooseneck snare, an additional movie file shows this in more detail (see Additional file 1). After the through-and-through guidewire was controlled, a 55 cm 7 F (COOK) sheath was advanced up to the level of bifurcation of the abdominal aorta through the left guidewire. Subsequently, an 0.018-inch V-18 guidewire (Boston Scientific Co.) was inserted via the right sheath and guided to the left femoral artery. Then, a 0.035-inch Lunderquist guidewire (COOK) was advanced into the thoracic aorta from the long sheath of the left femoral artery. Two 0.018-inch V-18 guidewires (Boston Scientific Co.) were used in the left sheath.

A gentle upward bilateral pull was maneuvered using a ‘‘cheese wire’’ technique to shear the flap of the mid-abdominal aorta from the distal tear of the left common iliac artery dissection up to the proximal end (Fig. 2). This operation was performed cautiously under the guidance of fluoroscopy to ensure that the extension of the intimal dissection did not occur. Next, angiography confirmed the formation of a suitable fenestration hole that provided access to the right femoral artery graft to enter the true lumen of the abdominal aorta (Additional file 1). A 28–30-mm cuff (Aortic Extender Endoprosthesis PLA280300, W. L. Gore & Associates Inc., Flagstaff, AZ, USA) was deployed at the proximal tear below the level of the renal artery to establish a normal landing zone and repair the proximal tear. A Hercules™ bifurcated stent graft system (Hercules™ HBB26-14-160; Microport, Shanghai) was introduced into the true lumen from the left femoral artery. An extender limb (Hercules™ HBL16-14-120) was placed from the right femoral artery. The coda balloon was used to expand the proximal end of the main stent and the stent-graft junction (Additional file 2).

**Fig. 3:**
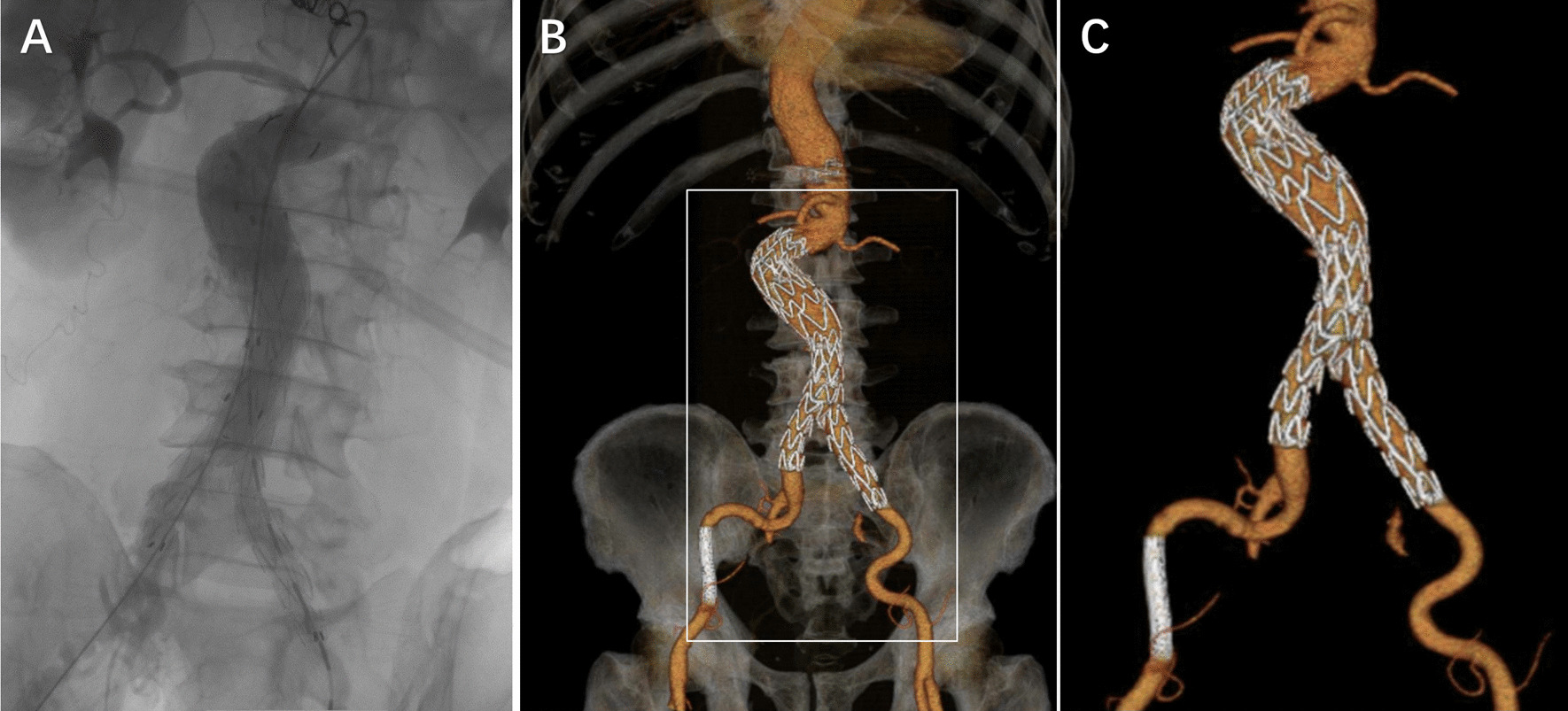
Postoperative digital subtraction angiography and three-dimensional reconstruction image. **A** Three stent grafts are placed for the normal perfusion of the common iliac arteries. **B** The abdominal aortic dissection is eliminated. **C** Postoperative three-dimensional reconstruction of the abdominal aortic dissection

**Fig. 4:**
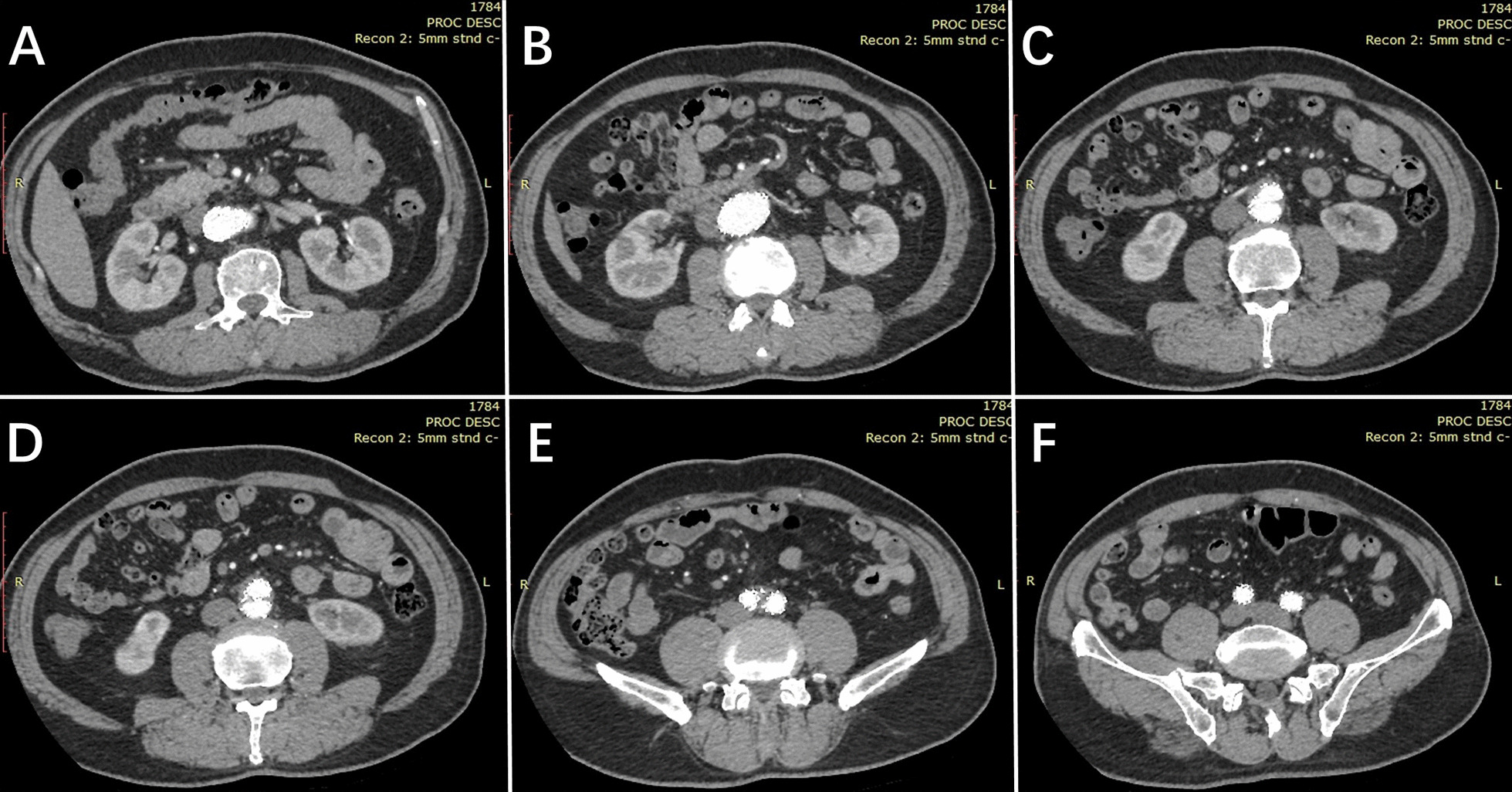
Postoperative axial-view surveillance computed tomography scans. **A** Abdominal aortic stent graft at the proximal end. **B** Normal perfusion of the left renal artery. **C** Normal perfusion of the right renal artery. **D** Bilateral common iliac artery stent grafts at the proximal end. **E** Normal perfusion in the bilateral common iliac artery stent grafts. **F** Bilateral common iliac artery stent graft at the distal end

Angiography confirmed the patency of the bilateral common iliac arteries and adequate visceral perfusion with no evidence of endoleak or arterial rupture (Figs. 3 and 4). The patient continued to take dual antiplatelets (such as aspirin, 100 mg, qd and clopidogrel, 75 mg, qd) for 12 months after this intervention. The patient had an uneventful postoperative course and was discharged on day 4 after the operation. At 6-months follow-up, no complaints of the lower limb ischemia were presented. Postoperative CTA demonstrated good stent-graft expansion and abdominal aortic dissection, excluding the patent perfusion of bilateral common iliac arteries; also, no endoleak was detected.

## Discussion and conclusions

The open surgery of type B aortic dissection is still associated with significant morbidity and mortality [6]. The Aorto-Uni-Iliac (AUI) stent graft-mediated endovascular abdominal aortic aneurysm repair (EVAR) with a femorofemoral bypass is considered as an alternative strategy; however, it is associated with a remarkable incidence of graft occlusion and stenosis of the contralateral internal iliac artery [7]. The “cheese wire” maneuver is used to fenestrate an intimal flap as it alleviates malperfusion in aortic dissection (Table 1). Also, the “cheese wire” technique is a long fenestration technology that could be accomplished endovascularly using a tension guidewire. This might decrease the risk of rupture or stent graft-induced new entry in comparison to balloon angioplasty or catheter rupture of the dissection septum. Although this technology is hazardous and risky, it is preferred in some cases. Herein, we presented a special case, wherein “cheese wire” was used in a reverse manner. Under normal circumstances, the “cheese wire” technique is always applied by directly pulling the guidewire. However, in this case, we used a catheter to push up, which resulted in a successful split of the endometrial flap.

**Table 1:** “Cheese wire” literature review

Author/year	Patient age (years)	Diagnosis	Follow–up time (months)	Result
Bolia et al. 1990	47–86	Occlusions of the femoro-popliteal segment	6	37 (84%) were either asymptomatic or improved
Watkinson et al. 2009 [9]	65	Common iliac occlusion	6	Asymptomatic and a normal right femoral pulse
Sebastian et al. 2011	60	Acute thoracico–abdominal type B dissection	–	No ischemic symptoms of the right leg
	69	Acute thoracico–abdominal type B dissection	–	No symptoms
	70	Iatrogenic type A dissection	–	Death
	60	Chronic thoracico–abdominal type B dissection	–	No symptoms
Jun Tashiro et al. 2013 [2]	70	Abdominal aortic aneurysm and chronic type B aortic dissection	3	No symptoms
Brant et al. 2015	57	Chronic residual chronic residual	8	No symptoms
Hozan et al. 2018	53	Complicated type A dissection	–	Death
Jordan et al. 2018	65	Acute aortic type A dissection	1	No symptoms and no endoleak
Iwakoshi et al. 2019	47	Loeys–Dietz syndrome, aortic arch aneurysm, and chronic Stanford type B aortic dissection	24	Type Ib endoleak and abdominal aorta repair was performed
	75	Proximal descending thoracic aorta and true lumen collapse	24	Type III endoleak and TEVAR was performed
	49	Type A aortic dissection	16	A stable descending aortic aneurysm with no endoleak
Current	62	Abdominal aortic type B dissection	6	No symptoms

This patient had symptoms of acute right iliac artery occlusion, such as severe pain in the right lower extremity and no pulse. Previous studies demonstrated the feasibility of using intravascular fenestration of dissection flap for treating poor perfusion secondary to acute aortic dissection [8–10]. Challenges were faced in treating the iatrogenic abdominal aortic dissection of the right iliac artery, which could be attributed to the fact that the location of the proximal aortic dissection fissure is unknown. The distal tear of the aortic dissection is located in the left common iliac artery, and the blood flow to the right common iliac artery originates from the false lumen. The catheter cannot be returned to the true lumen in the aortic dissection using conventional methods from the right access. Although the fenestration of the inner flap under intravascular ultrasound guidance of the vessel should be considered, it is limited by the equipment and hardware conditions, and hence, the use of “cheese wire” technique was chosen to supply the distal true lumen entry of the aortic artery from right femoral and restore the right lower extremity reperfusion promptly. The guidewire was snared within the false lumen from the distal tear after puncturing through the right femoral artery. Finally, the distal aortic dissection fissure was found according to the DSA, following which, the guidewire was transported to the true lumen. Accordingly, another capture guidewire was required to grab the sheath and guide it to the release position for stent-graft reconstruction. The operator should be ready for such possibility as the aorta might tear and rupture when conducting this interventional procedure, and rescue maneuvers such as rewiring, placement of covered stents to jail the occluding flap, or even urgent open conversion should be conducted if needed.

This would establish the true-false-true through wire access by maintaining the Lunderquist wire and sheath access to the true lumen on both sides. Completion angiography demonstrated excellent technical results with no evidence of endoleak and a satisfactory apposition of the graft to the aortic wall at the proximal neck. Also, the 6-month follow-up did not show an endoleak on CT angiography, and the patient reported to be feeling well. Taken together, the cheese-wire technique is a useful maneuver that facilitates endovascular arterial dissection repair. Adequate proximal or distal fixation could be conducted by endovascular fenestration. Nonetheless, sufficient experience is necessary for interventional therapy; also, total percutaneous approach is considered feasible for treating some sophisticated aortic dissections.

## Supplementary information

**Additional file 1.** The procedure of reverse “cheese wire” endovascular fenestration repair.

**Additional file 2.** Angiography confirmed the patency of the bilateral common iliac arteries and adequate visceral perfusion.

## Data Availability

All data generated or analyzed during this manuscript are included in this published article.

## Abbreviations

CWFER: “Cheese wire” fenestration endovascular repair
CTA: Computed tomography angiography
LMWH: Low molecular weight heparin
AUI: Aorto-Uni-Iliac
EVAR: Endovascular abdominal aortic aneurysm repair

## References

[1] Yang B, Rosati CM, Norton EL (2018). Endovascular Fenestration/Stenting First Followed by Delayed Open Aortic Repair for Acute Type A Aortic Dissection With Malperfusion Syndrome. Circulation.

[2] Tashiro J, Baqai A, Goldstein LJ (2014). “Cheese wire" fenestration of a chronic aortic dissection flap for endovascular repair of a contained aneurysm rupture. J Vasc Surg.

[3] Kamman AV, Yang B, Kim KM (2017). Visceral Malperfusion in Aortic Dissection: The Michigan Experience. Semin Thorac Cardiovasc Surg.

[4] Nordon IM, Hinchliffe RJ, Loftus IM (2011). Management of acute aortic syndrome and chronic aortic dissection. Cardiovasc Intervent Radiol.

[5] Erbel R, Aboyans V, Boileau C (2014). 2014 ESC Guidelines on the diagnosis and treatment of aortic diseases: Document covering acute and chronic aortic diseases of the thoracic and abdominal aorta of the adult. The Task Force for the Diagnosis and Treatment of Aortic Diseases of the European Society of Cardiology (ESC). Eur Heart J.

[6] Conway AM, Qato K, Mondry LR (2018). Outcomes of thoracic endovascular aortic repair for chronic aortic dissections. J Vasc Surg.

[7] Hossain S, Steinmetz OK, Corriveau MM (2016). Patency of the contralateral internal iliac artery in aortouni-iliac endografting. J Vasc Surg.

[8] Kos S, Gurke L, Jacob AL (2011). A novel fenestration technique for abdominal aortic dissection membranes using a combination of a needle re-entry catheter and the “cheese-wire”technique. Cardiovasc Intervent Radiol.

[9] Watkinson AF (2009). A novel “cheese wire” technique for stent positioning following difficult iliac artery subintimal dissection and aortic re-entry. Cardiovasc Intervent Radiol.

[10] Wuest W, Goltz J, Ritter C (2011). Fenestration of aortic dissection using a fluoroscopy-based needle re-entry catheter system. Cardiovasc Intervent Radiol.

